# Are Linguistic and Motricity domains intertwined in Schizophrenia? A preliminary analysis.

**DOI:** 10.1192/j.eurpsy.2023.597

**Published:** 2023-07-19

**Authors:** F. Magnani, N. Fascendini, V. Lucarini, C. Marchesi, M. Tonna

**Affiliations:** ^1^Medicine and Surgery, Unit of Neuroscience, Parma University Hospital, parma, Italy; ^2^Institute of Psychiatry and Neuroscience of Paris (IPNP), Université Paris Cité, Paris, France; ^3^Department of Mental Health, Local Health Service, Parma, Italy

## Abstract

**Introduction:**

The disruption of minimal Self is believed to be a core element of Schizophrenia and intimately connected to a disruption of bodily self, which in turn leads to impairments in intersubjectivity dimension. Motor abnormalities have been associated to Schizophrenia since the early conceptualization of the disorder, as well as inefficient body-related multisensory integration processes are considered nowadays a plausible origin of disembodied Self. In particular, there is evidence for significant abnormalities in Peripersonal Space (PPS) extension in Schizophrenia patients. PPS is the plastic sector of space immediately surrounding our body, whose coherent representation is based on efficient body-related multisensory integration processes. With a specific experimental task based on multisensory integration processing, we estimated PPS size and PPS boundary’s demarcation in 27 Schizophrenia patients, confirming a narrower PPS size and weaker bodily boundary in patients, thus paving the way for a deeper investigation of the mechanisms underlying the disruption of bodily self (Ferroni et al., Schziophr.Bull.2022, 5 1085-1093). We suggest that disembodiment might be responsible for the loss of the immediate linkage between Self and others (“intercorporeality”), so linking the disruption of the corporeal dimension to specific anomalies of intersubjectivity in Schizophrenia patients. Since language is one of the most important instrument through which intersubjectivity unfolds, it is intriguing to hypothesize a connection between language and multi-sensory processing.

**Objectives:**

Therefore, the present study was aimed at investigating possible correlations between patients’ motor impairments in multi-sensory integration processes and their alterations in language and communicative interactions.

**Methods:**

Twenty-five outpatients were recruited in an experimental task investigating PPS extension; they were administered the Scale for the Assessment of Thought, Language and Communication (TLC) and the Clinical Language Disorder Rating Scale (CLANG).

**Results:**

Our data showed significant correlations between TLC and CLANG total scores and PPS size, with narrower PPS size for more severe formal thought disorders and higher language and communication impairments.

**Conclusions:**

Our preliminary results seem to confirm the presence of a link between language impairment and multi-sensory processing, suggesting that bodily and linguistic disorganization may have a common origin which has yet to be explored in depth. Future research is needed to identify linguistic and motor endophenotypic patterns, potentially intertwined with each other, capable of early predicting Schizophrenia development and thus usable as early diagnostic tools.

**Disclosure of Interest:**

None Declared

